# CD161 Expression on Mucosa-Associated Invariant T Cells is Reduced in HIV-Infected Subjects Undergoing Antiretroviral Therapy Who Do Not Recover CD4^+^ T Cells

**DOI:** 10.20411/pai.v2i3.136

**Published:** 2017-08-07

**Authors:** Michael L. Freeman, Stephen R. Morris, Michael M. Lederman

**Affiliations:** 1 Center for AIDS Research, Division of Infectious Diseases and HIV Medicine, Department of Medicine, Case Western Reserve University/University Hospitals Cleveland Medical Center, Cleveland, Ohio; 2 Louis Stokes Cleveland VA Medical Center, Cleveland, Ohio

**Keywords:** HIV, Immune Failure, MAIT cells

## Abstract

**Background:**

Mucosa-associated invariant T (MAIT) cells are a recently identified class of innate-like T cells that are involved in the mucosal immune response. MAIT cells are characterized by expression of TCR Vα7.2 and CD161. In HIV infection, there is a profound early loss of MAIT cells from the circulation that never fully recovers, even after prolonged viral control with antiretroviral therapy (ART).

**Methods:**

We analyzed PBMCs from fresh whole blood from HIV-negative or ART-treated HIV-positive donors with full (Immune Success) or impaired (Immune Failure) CD4^+^ T- cell recovery by flow cytometry for T-cell markers, TCR Vα7.2, and CD161. The PBMCs were cultured with or without TCR-mediated stimulation, and CD161 expression was assessed on Vα7.2^+^ T cells. Interferon-γ (IFNγ) production was assessed by intracellular cytokine staining.

**Results:**

We found a decrease in the percentage of CD3^+^ T cells that expressed CD161 and the percentage of Vα7.2^+^ T cells that expressed CD161, in HIV-infected individuals. We also found a significant increase in the percentage of T cells that were Vα7.2^+^CD161- in immune failure compared to controls, accompanied by an increase in the percentage of Vα7.2^+^CD161- T cells that express CD8^+^ in donors with immune failure, but not immune success. After TCR stimulation in vitro, Vα7.2^+^ T cells reduced expression of CD161, yet Vα7.2^+^ CD161- cells from immune failure donors retained the ability to express IFNγ on stimulation.

**Conclusions:**

Our findings suggest that in immune failure patients, the reduction in peripheral MAIT cells is due, at least in part, to a loss in CD161 expression, and is not merely the result of trafficking into mucosal tissues or cell death. These CD161- cells retain their function.

## INTRODUCTION

Infection with Human Immunodeficiency Virus (HIV) is well controlled in patients who adhere to a regimen of combination antiretroviral therapy (ART), with most patients achieving viral suppression. The majority of patients on ART recover CD4^+^ T-cell numbers to over 500 cells/μL (a population termed immune responders or immune success), but about a third of ART-treated individuals fail to recover CD4^+^ T-cell numbers to over 350 cells/μL (a population termed immune non-responders or immune failure) [1–4]. Patients with immune failure are most often male, older, and have lower nadir CD4^+^ T-cell counts [3].

Not only do immune failure patients have lower peripheral blood CD4^+^ T-cell counts, they also have evidence of chronic inflammation, breakdown of the gut mucosal barrier, and elevated risk of co-morbidities such as cardiovascular disease [5, 6]. Plasma levels of the proinflammatory cytokine interleukin-6 (IL-6), the T-cell proliferation driver IL-7, the soluble form of the lipopolysaccharide (LPS) co-receptor CD14, and the fibrin degradation product D-dimer are all elevated in immune failure patients [3, 7]. In addition, both CD8^+^ and CD4^+^ T cells in the circulation of immune failure donors have a more activated, differentiated phenotype than in immune success individuals [3, 8].

Another lymphocyte type that is affected during HIV infection is the mucosa-associated invariant T (MAIT) cell. MAIT cells are a recently identified T-cell population that has features of both innate and adaptive immunity. They are characteristically defined by expression of the T-cell receptor (TCR) invariant variable α-chain 7.2 (Vα7.2)-Jα33 [9] and high expression of the natural killer (NK) cell marker CD161 [10]. In HIV-uninfected individuals, MAIT cells can comprise around 10% of all CD3^+^ T cells in the circulation and are further enriched in the liver and in mucosal tissues [9, 11]. Traditional CD8^+^ or CD4^+^ T cells recognize peptides presented by major histocompatibility complex (MHC) molecules I or II, respectively, but MAIT cells are specific for components of the riboflavin (vitamin B_2_) biosynthetic pathway presented by the MHC-I-related (MR) molecule MR1 [9, 12–15]. Because the riboflavin biosynthetic pathway is present in many bacteria and some fungi, this specificity allows MAIT cells to respond to microbial infection [12–14]. The MAIT cells can kill targets in a granzyme-dependent manner [16, 17] and they produce the effector cytokines interferon-γ (IFNγ) and tumor necrosis factor (TNF) as well as IL-17 and IL-22, which are 2 key cytokines in the regulation of intestinal immunity and maintenance of the gut mucosal barrier [18–20].

MAIT cells are depleted from the periphery soon after HIV infection. While numbers of MAIT cells in the gut reconstitute following ART administration, circulating MAIT-cell populations do not recover [21, 22]. There is no consensus on the functionality of peripheral MAIT cells remaining in people infected with HIV, and differences in the methods of stimulation and patient populations may explain the discordant findings [21, 23].

Loss of MAIT cells is not likely to be a consequence of direct virus infection [22] and could be due to recruitment to peripheral tissues, activation-induced apoptosis, or down-regulation of CD161. The MAIT cells express many chemokine receptors such as CCR5 and CCR6 that direct them to sites of mucosal inflammation [11, 21, 22]. There is also evidence that MAIT cells are susceptible to apoptosis following activation [22, 24]. It is less clear if the loss of peripheral MAIT cells in HIV infection could be due to a phenotypic change in the cells that hide them from conventional identification [25, 26]. To this end, it has been demonstrated that a Vα7.2^+^CD161- population accumulates in individuals with chronic untreated HIV infection, and this subset retains surface receptors and transcription factors important to MAIT-cell function [21, 23]. Whether this population represents CD161-negative “ex-MAIT” cells or a newly generated Vα7.2^+^ population is controversial, and has never been specifically examined in ART-treated individuals with impaired CD4^+^ T-cell recovery.

## METHODS

**Donors:** This work was approved by the Institutional Review Board at University Hospitals Cleveland Medical Center (#01-98-55) in accordance with the guidelines of the Declaration of Helsinki. With written informed consent, whole blood was collected in EDTA Vacutainer tubes (BD Biosciences) from HIV-uninfected people (n = 27), and ART-treated HIV-infected people with undetectable viremia and either CD4^+^ T-cell recovery (Immune Success or Immune responders: CD4^+^ T-cell counts > 500 cells/μL)(n = 23) or incomplete CD4^+^ T-cell recovery (Immune Failure or Immune non-responders: CD4^+^ T-cell counts < 350 cells/μL)(n = 19). The CD4^+^ T-cell counts were determined in the hospital clinical laboratory by flow cytometry, and CD4/CD8 ratios were determined in the research laboratory by flow cytometry. Participant characteristics are shown in Table 1.

**Table 1.** Participant Characteristics.

	HIV-positive		Total	
	Immune Success	Immune Failure	HIV-positive	HIV-negative
N (male, %)	23 (78.3%)	19 (94.7%)	42 (85.7%)	27 (44%)
Age (y), Median (IQR)	52 (46–55)	53 (49–62)	53 (48–56)	30 (25–44)
Time of ART^a^ (y), Median (IQR)	9.71 (6.44–13.83)	10.13 (6.44–20.1)	9.96 (6.5–14.47)	NA
CD4^+^ (cells/uL), Median (IQR)	816 (625–1009)	270 (230–312)	475 (277–848)	NA
CD4/CD8 Ratio, Median (IQR)	1.08 (0.59–2.64)	0.49 (0.32–0.75)	0.75 (0.43–1.38)	1.84 (132–2.42)
CD4^+^ nadir (cells/uL), Median (IQR)	140 (39–258)	24 (6–66)	67 (9.5–176.5)	NA

^a^ART, antiretroviral therapy

**Tissue processing:** PBMCs were purified by centrifugation over a Ficoll-Hypaque (GE Health-care) cushion and cultured in RPMI 1640 medium supplemented with 10% fetal bovine serum (FBS; Gibco), 1% penicillin/streptomycin (Gibco), and 1% L-glutamine (Gibco) at 37°C and 5% CO_2_.

**Flow Cytometry:** Lymphocytes were identified by forward and side scatter, and cell identity was assessed using fluorochrome-conjugated antibodies anti-CD3 (clone SK7; eBioscience), anti-CD4^+^ (RPA-T4; BD), anti-CD8 (RPA-T8; BD), anti-CD161 (DX12; BD), and anti-TCR Vα7.2 (3C10, BioLegend). Viable cells were gated using Live/Dead Yellow or Live/Dead Aqua viability dyes (Invitrogen) according to the manufacturer's instructions. Cells were stained for 20 minutes in the dark at room temperature, washed, and fixed in PBS containing 2% formaldehyde. All samples were acquired on LSRII or LSRFortessa flow cytometers (BD). Cell division was assessed by labeling PBMCs with 5(6)-carboxyfluorescein diacetate succinimidyl ester (CFSE) (Molecular Probes) for 10 minutes at 37°C. Staining was quenched by the addition of FBS for 5 minutes on ice. Cells were then washed and cultured as described.

For detection of intracellular cytokines, cells were stimulated with 50ng/mL soluble anti-CD3 (HIT3a; BD) and 3μg/mL soluble anti-CD28 (CD28.2; BD) for 24 hours at 37°C and 5% CO_2_, in the presence of brefeldin A (GolgiPlug; BD) for the final 6 hours of stimulation. After stimulation, cells were washed, stained with viability dye and antibodies to surface antigens, then fixed and permeabilized using the Cytofix/Cytoperm kit (BD) and stained intracellularly with fluoro-chrome-conjugated antibody to IFNγ (B27, BD).

**Statistics:** We compared continuous variables using the Mann-Whitney *U* test or the Kruskal-Wallis test with Dunn's correction for multiple variables. Correlations were determined using a nonparametric Spearman test. *P* values ≤ 0.05 were considered statistically significant.

## RESULTS

### Participant Characteristics

PBMCs were harvested from whole blood from HIV-uninfected donors, or ART-treated HIV-infected donors with CD4^+^ T-cell recovery (immune success: > 500 CD4^+^ T cells/μL) or poor CD4^+^ recovery (immune failure: < 350 CD4^+^ T cells/μL). Participant characteristics are shown in Table 1. Although our healthy control cohort was not well-matched to the HIV-infected groups, we have found that neither age (HIV- *r* = -0.2194, *P* = 0.3393; HIV^+^ *r* = -0.1623, *P* = 0.4093; Spearman analysis) nor sex (HIV- *P* = 0.7675; HIV^+^ *P* = 0.2038; Mann-Whitney) had an effect on MAIT cell proportion; thus we are confident in the comparisons in this study.

### Reduction of CD161^+^ cells

**Figure 1.**
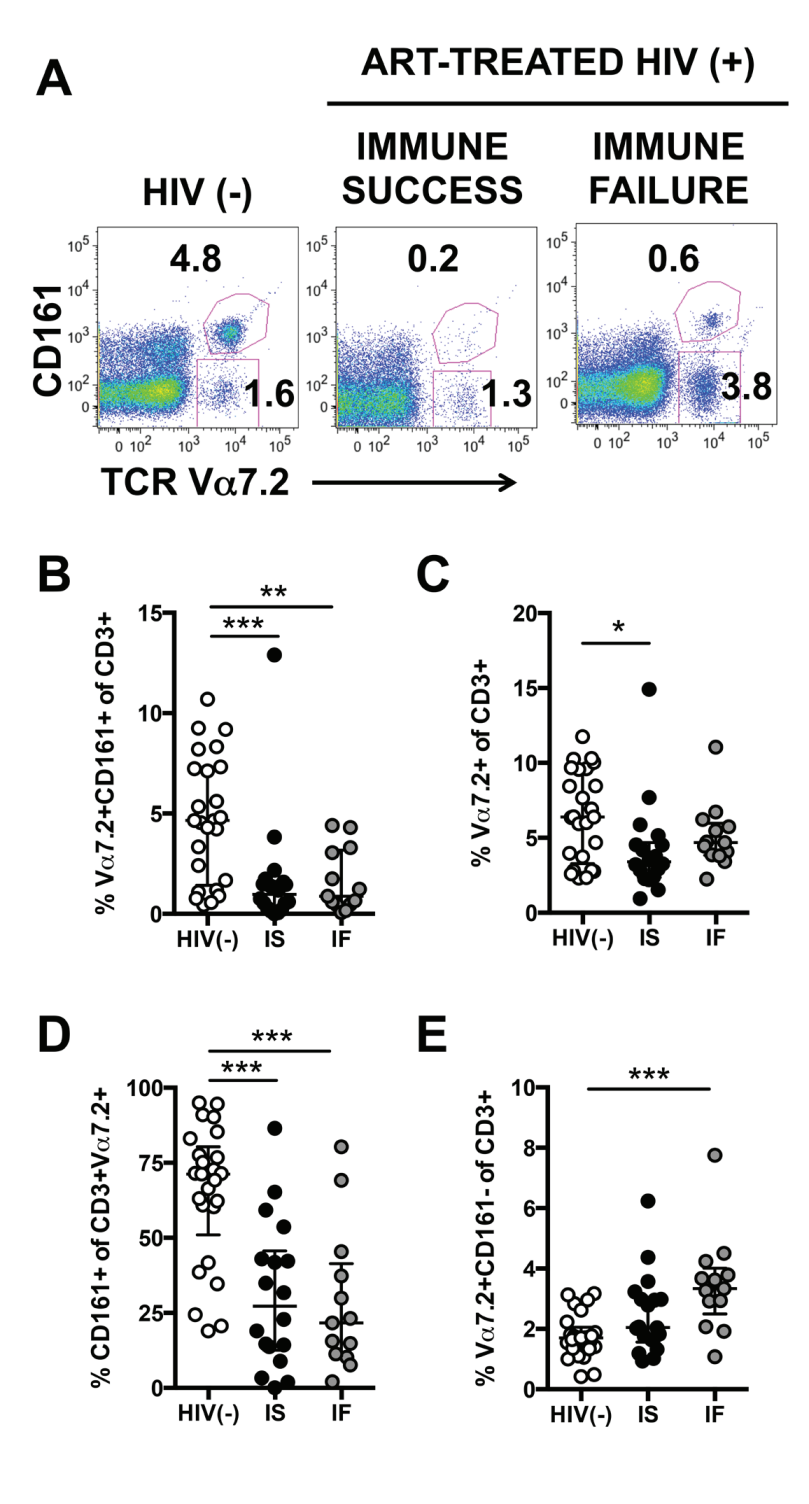
**Loss of Vα7.2^+^CD161^+^ Cells in ART-Treated HIV Infection.** (A) Representative plots show CD161 and TCR Vα7.2 expression on CD3^+^ cells from HIV-uninfected or ART-treated HIV-infected donors. (B) The percentage of CD3^+^ cells that are Vα7.2^+^CD161^+^ (Kruskal-Wallis test). (C) The percentage of CD3^+^ cells that are Vα7.2^+^ (Kruskal-Wallis test). (D) The percentage of CD3^+^Vα7.2^+^ cells that are CD161^+^ (Kruskal-Wallis test). (E) The percentage of CD3^+^ cells that are Vα7.2^+^CD161- (Kruskal-Wallis test). *P ≤ 0.05; **P ≤ 0.01; ***P ≤ 0.001. ART, antiretroviral therapy; IS, Immune Success; IF, Immune Failure.

MAIT cells are often characterized by their co-expression of the NK cell marker CD161 and TCR Vα7.2. The MAIT cells are CD3^+^ and are most often CD8^+^, but they can also be CD4^+^ or double negative (DN; CD4-CD8-)[10, 27]. Therefore, we gated on total live CD3^+^ cells and examined CD161 and Vα7.2 expression in PBMCs from healthy control, immune success, or immune failure participants by flow cytometry. Representative dotplots are shown in Figure 1A. As expected, the percentage of CD3^+^ cells that were Vα7.2^+^CD161^+^ was significantly reduced in HIV-infected donors (Figure 1B). This was not due to an overall loss of Vα7.2^+^ cells, because even though total Vα7.2^+^ cells were also reduced (Figure 1C), the percentage of Vα7.2^+^ cells that were CD161^+^ was further decreased (Figure 1D). Intriguingly, in immune failure subjects, the percentage of CD3^+^ T cells that were Vα7.2^+^CD161- was actually increased, compared with the percentages in both immune success subjects and healthy controls (Figure 1E). Expression of CD161 by Vα7.2^+^ cells was equivalent after surface and intracellular staining in all 3 groups of donors (data not shown), verifying that loss of CD161 was not due to receptor internalization.

### Accumulation of CD8^+^Vα7.2^+^CD161- cells

**Figure 2.**
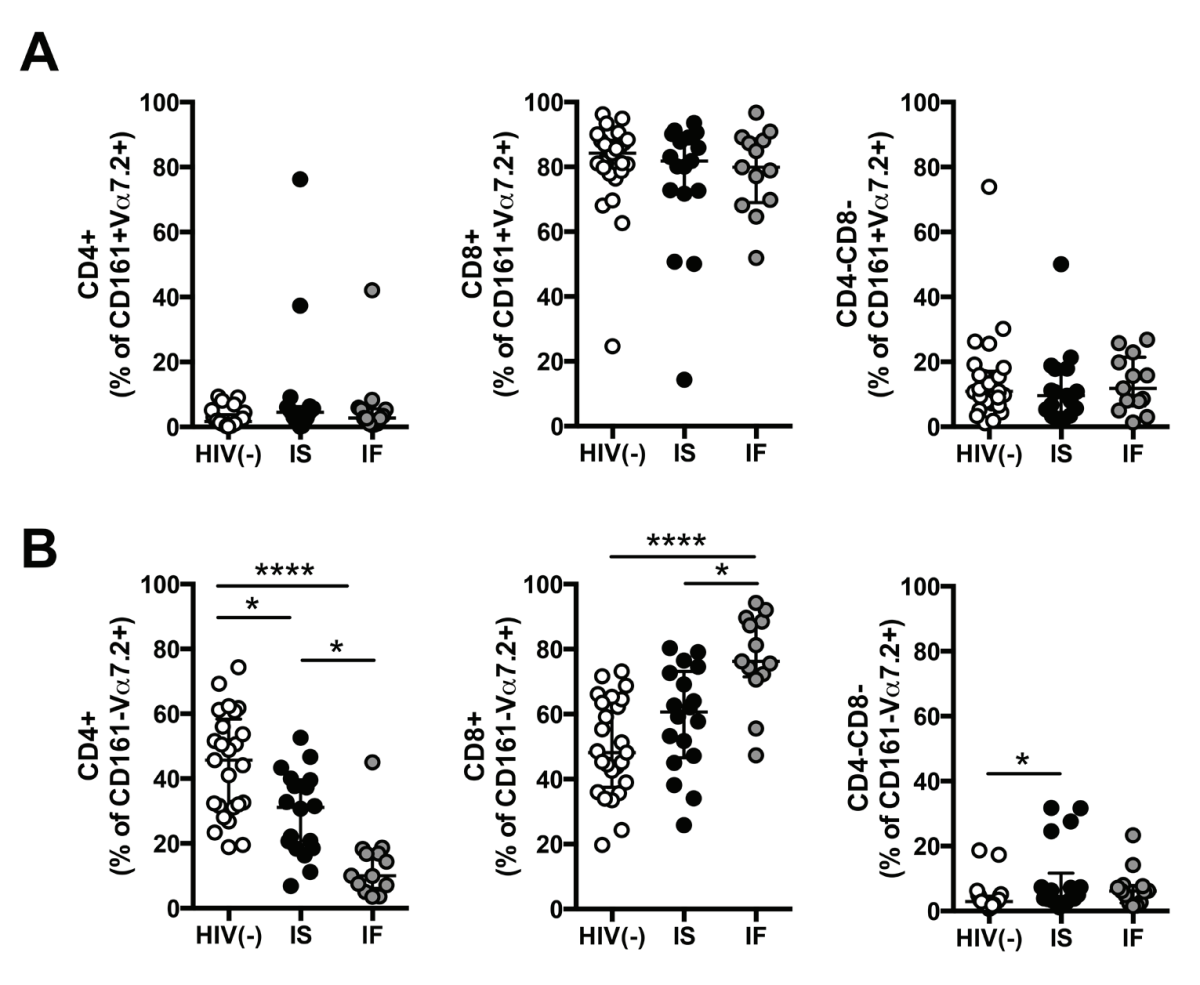
**Accumulation of CD8^+^Vα7.2^+^CD161- Cells in Immune Failure Patients.** (A, left) The percentage of CD3^+^Vα7.2^+^CD161^+^ cells that are CD4^+^. (A, center) The percentage of CD3^+^Vα7.2^+^CD161^+^ cells that are CD8^+^. (A, right) The percentage of CD3^+^Vα7.2^+^CD161^+^ cells that are CD4-CD8-. All comparisons were *P* > 0.05 using Kruskal-Wallis test. (B, left) The percentage of CD3^+^Vα7.2^+^CD161- cells that are CD4^+^ (Kruskal-Wallis test). (B, center) The percentage of CD3^+^Vα7.2^+^CD161^+^ cells that are CD8^+^ (Kruskal-Wallis test). (B, right) The percentage of CD3^+^Vα7.2^+^CD161^+^ cells that are CD4-CD8- (Kruskal-Wallis test). *P ≤ 0.05; ****P ≤ 0.000. IS, Immune Success; IF, Immune Failure.

These observations led us to wonder if the loss of CD161^+^ cells could be due not only to cell death or traffic out of the circulation into the periphery, but also to a downregulation of the CD161 molecule itself, particularly in immune failure donors. To investigate this possibility, we examined the proportions of CD161^+^ and CD161- CD3^+^ Vα7.2^+^ cells that were CD4^+^, CD8^+^, or double negative (Figure 2). Cells that were CD161^+^ had remarkably similar distributions, regardless of the donor source—they were mostly CD8^+^, with a few double negatives and hardly any CD4^+^ cells (Figure 2A). In healthy controls, about half the number of Vα7.2^+^CD161- cells were CD4^+^, and this proportion was decreased in HIV-infected patients, particularly in immune failures (Figure 2B). Reciprocally, in the immune failure group, the proportion of Vα7.2^+^CD161- cells that were CD8^+^, but not double negative, was significantly increased—suggesting that there was a specific accumulation of CD8^+^CD161- cells within the Vα7.2^+^ population in the absence of complete CD4^+^ T-cell recovery. This change appears to be specific for CD8: it is unlikely to be due to overall poor CD4^+^ recovery, as that would be expected to affect the percentages of both CD8 and DN cells equally, which did not occur for immune failure donors.

### Loss of CD161 with stimulation

**Figure 3.**
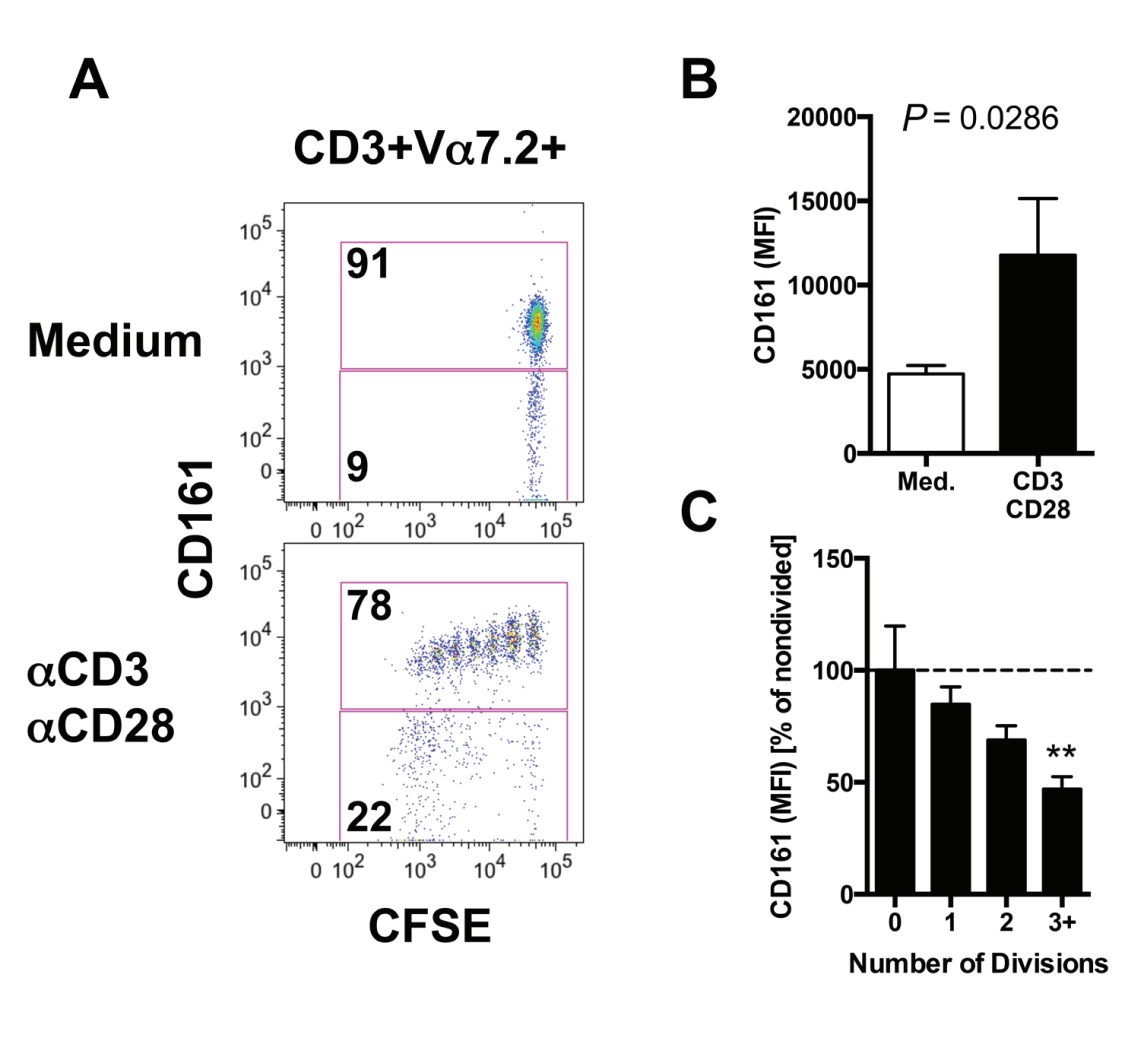
**Cell Division and Reduction in Surface CD161 Expression Following TCR-Mediated Stimulation.** (A) Representative plots show CD161 expression and CFSE dilution (proliferation) in CD3^+^Vα7.2^+^ cells following 4 days culture with control medium (Med.) or stimulating antibodies to CD3 (αCD3) and CD28 (αCD28). (B) The mean fluorescence intensity (MFI) of CD161 staining on undivided CD161^+^ cells as shown in (A) (n = 4; Mann-Whitney test). (C) The MFI of CD161 staining on CD161^+^ cells at each round of division normalized to non-divided cells (n = 4; Kruskal-Wallis test). **P ≤ 0.01.

Microbial translocation is a likely driver of the persistent inflammation observed in ART-treated HIV infection [5], and immune failure donors have evidence of elevated microbial products and inflammatory mediators in their circulation [3]. MAIT cells—which recognize products of microbial metabolism [13–15]—are therefore likely to encounter their antigens of interest at a greater level in the immune failure group. To model this situation in vitro we stimulated cultures of PBMCs from HIV-negative donors with anti-CD3 and anti-CD28, then measured cell division and CD161 expression on CD3^+^Vα7.2^+^ cells 4 days later (Figure 3A).

Interestingly, in undivided Vα7.2^+^CD161^+^ cells, TCR-mediated stimulation resulted in a significant upregulation of CD161 expression (Figure 3B). However, as the CD161^+^ cells divided, and with each subsequent division, the mean fluorescence intensity (MFI) of CD161 on the cell surface became lower (Figure 3C). Thus, MAIT cells reduce CD161 expression as they divide in response to TCR stimulation. This observation is consistent with the findings of Leeansyah *et al*, who demonstrated that Vα7.2^+^CD161- cells accumulate in the blood and rectal mucosa during chronic untreated HIV infection, and can be generated *in vitro* by bacterial stimulation [21].

### Vα7.2^+^CD161- CD8^+^ T cells retain function in immune failure, but not immune success or HIV-negative donors

**Figure 4.**
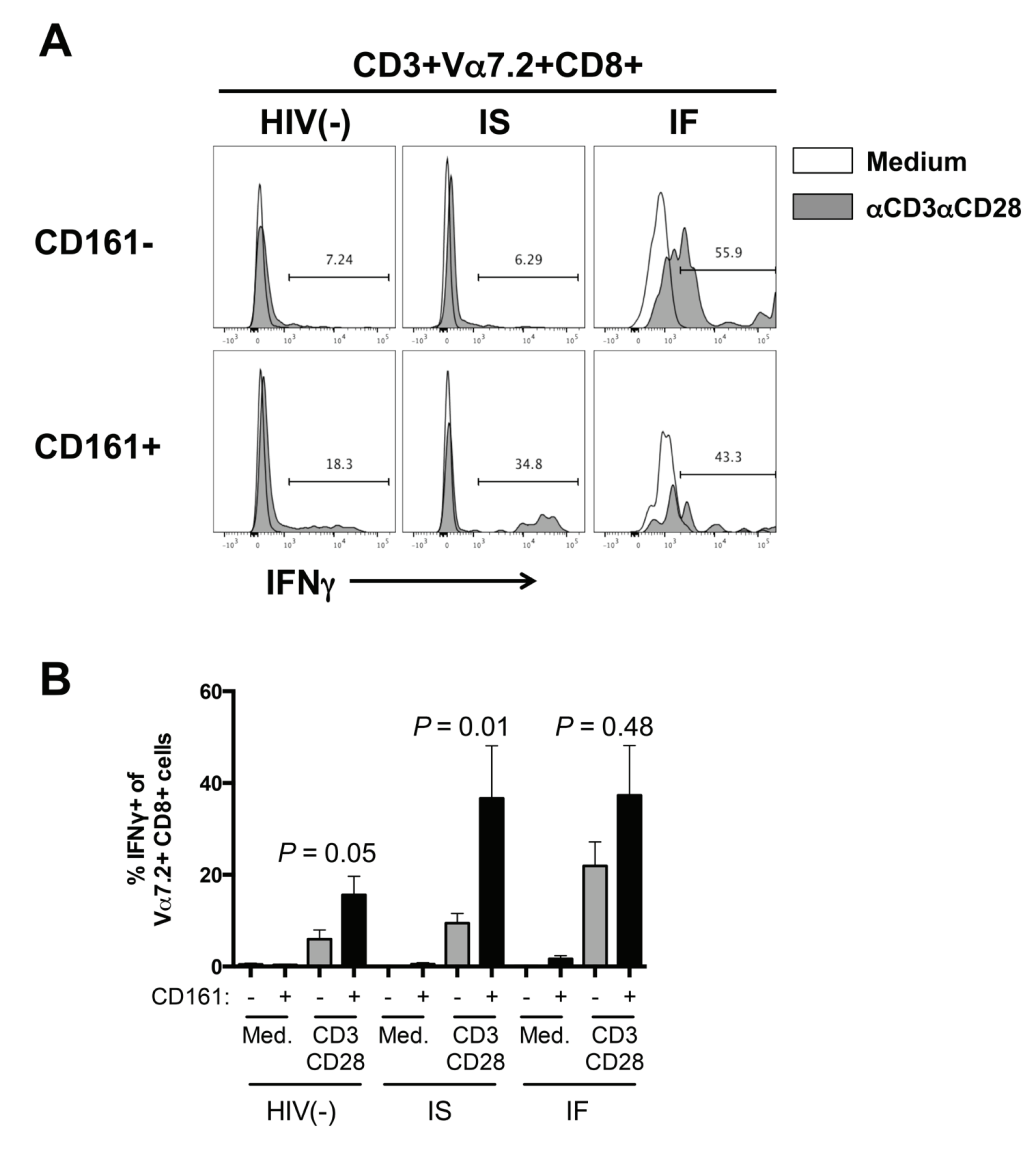
**Sustained IFNγ Production by Vα7.2^+^CD161- Cells From Immune Failure Donors.** (A) Representative histograms show intracellular IFNγ synthesis by CD3^+^Vα7.2^+^CD8^+^ cells that are CD161-or CD161^+^ following 24 hours in culture with control medium or stimulating antibodies to CD3 (αCD3) and CD28 (αCD28). Numbers indicate the percentage of CD8^+^ cells that express IFNγ. (B) The percentage of CD3^+^Vα7.2^+^CD161-CD8^+^ (gray bars) or CD3^+^Vα7.2^+^CD161^+^CD8^+^ (black bars) cells that are IFNγ^+^ (n = 5-6; Mann-Whitney test).

To determine if there was a functional difference in the Vα7.2^+^ cells that were CD161^+^ or CD161-, we stimulated the TCR of PBMC preparations from each donor group for 24 hours and measured the cells for synthesis of IFNγ, a key cytokine produced by MAIT cells, particularly in response to microbial antigens [11, 13, 21, 22]. In all donor groups, Vα7.2^+^CD161^+^ cells were nearly all CD8^+^, and some of these cells produced IFNγ (Figure 4A,B). In HIV-uninfected and immune success donors, there was a significantly lower proportion of CD8^+^ T cells producing IFNγ in the Vα7.2^+^CD161- subset compared to the CD161^+^ subset (Figure 4A,B). In contrast, Vα7.2^+^CD161-CD8^+^ cells from immune failure donors partially retained the ability to synthesize IFNγ as there was no significant difference (compared to CD161^+^ cells) in the proportion of CD8^+^Vα7.2^+^ cells producing IFNγ. These data are therefore consistent with a reduction in CD161 expression on MAIT cells from immune failure donors leading to an increased population of Vα7.2^+^CD161-cells which retain functionality.

## DISCUSSION

The exact role of CD161 is not clear. It appears to function as a costimulatory receptor on CD8^+^ T cells and NK cells, but may have opposite effects depending on the cell type [28, 29]. The ligand for CD161, lectin-like transcript-1 (LLT1), is expressed on lymphocytes and activated antigen-presenting cells (APCs) [28–30]. Although CD161 impairs IFNγ and cytotoxicity in NK cells, CD161 ligation enhances IFNγ expression in CD8^+^ T cells [29]. Consistent with the relationship between CD161 ligation and enhanced IFNγ production, Leeansyah *et al* did not observe IFNγ, TNF, or IL-17 production from the Vα7.2^+^CD161- population of untreated patients in response to overnight stimulation with fixed *E. coli* [21]. Here, we also observed significantly higher function in the Vα7.2^+^CD161^+^ subset as compared to the Vα7.2^+^CD161- subset in cells from HIV-uninfected and immune success donors; however, CD8^+^Vα7.2^+^CD161- T cells from immune failure donors partially retained the ability to express IFNγ in response to TCR stimulation.

What drives the loss of CD161^+^ MAIT cells? It is suspected that ART-treated patients who fail to recover CD4^+^ T cells (ie, immune failure patients) also have increased microbial translocation due to excessive gut permeability [6]. MAIT cells specifically recognize bacterial or fungal vitamin B_2_ metabolites presented to their TCR by the MHC-I-like molecule MR1 [13–15]. An increase in circulating bacterial antigens might stimulate the MAIT cells in the bloodstream to traffic into mucosal sites where they undergo activation-induced cell death or loss of surface CD161 expression due to excessive stimulation and/or replication. We have shown that after TCR stimulation, cell division corresponds with decreased surface expression of CD161 on MAIT cells.

Based on the absence of CD161 expression, a lack of MR1 tetramer binding [23], and previously shown poor functionality [21], some researchers have proposed that the Vα7.2^+^CD161- cells in chronic HIV infection are not like MAIT cells phenotypically or functionally [25]. On the other hand, Vα7.2^+^CD161- cells from individuals with chronic untreated HIV infection, but not un-infected controls, have been shown to co-express high levels of IL-18 receptor (IL-18R) and the transcription factor PLZF—both of which promote MAIT cell functionality [31–33]. Heightened expression of these factors in both CD161^+^ and CD161- subsets is likely to be biologically signifi-cant since HIV+ patients have higher circulating IL-18 levels than do uninfected controls [34, 35]

A likely determinant of why MAIT cells from immune success and immune failure are affected differently is *in vivo* cytokine exposure, as MAIT cells are susceptible to activation by several cytokines. For instance, the homeostatic cytokine IL-7 enhances MAIT-cell activation and cytokine production in response to TCR stimulation [36], and promotes MAIT-cell cytotoxicity by increasing stores of intracellular perforin and granzymes [37]. These effects could be particularly relevant in immune failure subjects who have elevated plasma IL-7 levels [7]. Similar results were seen with a combination of IL-1β and IL-23 [36]. Our group has shown that IL-1β protein is increased in the lymph node of ART-naive HIV-infected individuals [38]. Early IL-1β expression (prior to the initiation of ART) in the presence of systemic microbial antigens could potentially accelerate MAIT cell activation leading to exhaustion, dysregulation, and death, which is consistent with the observation that most CD161^+^ MAIT cell loss occurs during acute HIV infection [22]. As mentioned above, MAIT cells are highly enriched for expression of IL-18R [39], and IL-18 in combination with IL-12 induces IFNγ expression in MAIT cells even in the absence of TCR signals [31]. Indeed, long-term stimulation of MAIT cells by bacteria-laden APCs appears to be dependent on IL-12/IL-18 signals for optimal IFNγ induction [31]. Consistent with these observations, MAIT cells are activated in a TCR-independent manner by several human viral infections, and this activation is dependent on infection-induced cytokines including IL-12, IL-15, IL-18, and type I interferons [40].

The cytokine IL-15 may prove a particularly important mediator of MAIT-cell activation during HIV infection. Serum IL-15 levels have been significantly correlated to increasing HIV viremia, low CD4 T cell count, and markers of inflammation and coagulation such as D-dimer, sCD14, CRP, and sCD163 [41]. In addition, immune failure and untreated patients have increased PBMC-derived IL-15 production compared to immune success and HIV-uninfected donors [42]. We have recently demonstrated that IL-15 levels are elevated in the lymph nodes during viremic HIV infection [43], but there are no reports on the relationship between lymph node IL-15 levels and degree of immune reconstitution in HIV patients undergoing ART. In addition, IL-15-induced production of IL-18 by monocytes could activate MAIT cells, and IL-15 treatment induces the proliferation of Vα7.2^+^CD161^+^ cells (our unpublished observation).

It has been suggested that the numbers of mucosal MAIT cells are reduced at a lower rate than blood MAIT cells in HIV infection, possibly indicative of MAIT-cell traffic from the circulation into mucosal sites or enhanced survival of MAIT cells located in the mucosa [21, 26]. Notably, there is also a significant increase in the proportion of rectal mucosa T cells that are Vα7.2^+^ but CD161- [21]. Unlike in peripheral blood, the numbers of CD161^+^ cells in the colon recover in patients treated with ART that have sufficient CD4^+^ T-cell recovery [44], but it is not known if this colonic MAIT reconstitution is also observed in immune failure patients.

In HIV infection, a return of proper MAIT-cell numbers and functionality could potentially alleviate gut epithelial-barrier permeability, thereby reducing the translocation of microbial products and mitigating systemic inflammation. Therefore, it is of keen interest to explore methodology to recover MAIT-cell number in HIV infection. As noted above, there is some evidence that MAIT-cell function [21] and numbers in mucosal tissue [44] are restored after ART. Therapeutic administration of cytokines such as IL-2, IL-7, or IL-15 might trigger MAIT-cell proliferation or restore MAIT cell functionality, but could also induce HIV reactivation or exacerbate T-cell-driven immunopathology. Cytokine treatment might have to be coupled with blockade of IL-12/IL-18 to promote MAIT-cell proliferation while inhibiting activation. Alternatively, probiotics might alter the gut microbiota in such a way as to reduce MAIT-cell activation or induce their proliferation [45].

Although our data are consistent with a stimulation-induced loss of CD161 expression on Vα7.2^+^ MAIT cells during ART-treated HIV infection, particularly in immune failure subjects, we cannot completely rule out the possibility that Vα7.2^+^CD161- cells are not “ex-MAIT” cells, but rather an expansion of a separate cell population. This hypothesis has some merit, particularly because it has been shown that Vα7.2^+^CD161- cells do not bind an MR1 tetramer reagent in healthy individuals [46] or in chronic HIV infection [23]. Notably, both studies used frozen PBMC samples, and it is unknown if freezing affects MR1 tetramer binding in CD161- cells. Alternatively, Vα7.2^+^CD161- cells accumulating in chronic HIV infection do not express Ki-67, suggesting that these cells are not proliferating [21] and therefore not the result of expansion of a previously minor population, but rather the result of a phenotypic conversion from CD161^+^ cells. In any case, expansion of Vα7.2^+^CD161- CD8^+^ T cells is an intriguing possibility worthy of further study, particularly because activated memory CD8^+^ T cells expand in untreated HIV infection due to IL-15 signals [43], remain at elevated numbers for years after ART initiation, particularly in cytomegalovirus-seropositive subjects [47], and could contribute to non-AIDS-associated co-morbidities such as cardiovascular disease [48].

Future studies of immune failure patients should investigate if Vα7.2^+^CD161- cells can produce other conventional MAIT cell cytokines following TCR stimulation and whether the expansion of Vα7.2^+^CD161- cells is correlated to soluble markers of enhanced inflammation and coagulation (such as IFNγ, sCD14, D-dimer, sCD163, and CRP). Characterizing the functional capacity and maturation pathway of Vα7.2^+^CD161- cells (ie, whether they are “ex-MAIT” cells or not) might provide key insights into immunopathogenesis and the increased risk of adverse clinical events in immune failure subjects.
